# User-centered design of a personal-use exoskeleton: a clinical investigation on the feasibility and usability of the ABLE Exoskeleton device for individuals with spinal cord injury to perform skills for home and community environments

**DOI:** 10.3389/fnins.2024.1437358

**Published:** 2024-09-26

**Authors:** Franziska Nadorf, Mark Andrew Wright, Helena López-Matas, Erika Porras, Alfons Carnicero-Carmona, Cornelia Hensel, Steffen Franz, Norbert Weidner, Joan Vidal, Eloy Opisso, Rüdiger Rupp

**Affiliations:** ^1^Spinal Cord Injury Center, Heidelberg University Hospital, Heidelberg, Germany; ^2^Institut Guttmann, University Institute Attached to the Universitat Autònoma de Barcelona, Barcelona, Spain; ^3^Universitat Autònoma de Barcelona, Bellaterra, Cerdanyola del Vallès, Spain; ^4^Fundació Institut d’Investigació en Ciències de la Salut Germans Trias i Pujol, Badalona, Spain; ^5^ABLE Human Motion S.L., Barcelona, Spain

**Keywords:** spinal cord injury, lower limb, exoskeleton, feasibility, usability, personal use, home and community, user-centered design

## Abstract

**Introduction:**

The ABLE Exoskeleton has been tested to be safe and feasible for persons with spinal cord injury (SCI) to complete basic skills in clinical settings but has not been tested for use in home and community environments. A user-centered design process was employed to test the feasibility of the current ABLE Exoskeleton (designed for clinical use) for persons with SCI to perform the basic and advanced skills required for home and community environments, to gain crucial feedback for the development of a novel personal-use exoskeleton.

**Methods:**

In this prospective pretest-posttest quasi-experimental study across two SCI centers (Germany, Spain), in-and outpatients with SCI were included into a 22-session training and assessment protocol, utilizing the ABLE Exoskeleton. Feasibility and usability measures [level of assistance (LoA) for basic and advanced skills, donning/doffing-time and LoA] were recorded together with safety outcomes, and participant and therapist satisfaction with the device.

**Results:**

10 participants (44.4 ± 24 years), with SCI from C5 to T11, (American Spinal Injury Association Impairment Scale A–D) completed the study. In 209 sessions, six device-related adverse events (pain and skin lesions) were reported. Average total time for don and doff was 10:23 ± 3:30 min. Eight participants were able to complete don and doff with minimal assistance or less. Independence to carry out all skills in the device increased significantly for all participants (*p* < 0.05). Participants with chronic SCI required a significantly (*p* < 0.05) lower LoA for six of the nine advanced skills than those in the sub-acute phase.

**Discussion:**

This study shows that the ABLE Exoskeleton is safe, feasible and usable for people with SCI in respect to independent donning, doffing and performance of basic and advanced exoskeleton skills. The supervised exoskeleton use in the clinical environment was a highly valuable approach for identifying the challenging tasks and the necessary technological developments that need to be carried out for a personal-use exoskeleton, including a more independent sit-to-stand transition, faster speed of transitions between states and a richer display on the remote control for the user.

**Clinical trial registration:**

https://clinicaltrials.gov/study/NCT05643313.

## Introduction

In recent years, technology has evolved to be an important component within health-enhancing physical activity therapy programs for persons with Spinal Cord Injury (SCI), with robotic exoskeletons as one of the most notable technological developments (Tamburella et al., 2022; Miller et al., 2016). Wearable robotic exoskeletons have the potential to be used at home or in the community—not as a replacement for a wheelchair, but as an alternative device to enhance mobility and physical activity (Kandilakis and Sasso-Lance, 2021). Although many of these exoskeleton devices have been robustly trialed and tested within the clinical environment, there are only very few studies that have been able to show that this feasibility of use is transferable to the home or community setting. One of the key reasons for this is that exoskeletons initially developed for a clinical setting can often be found to be inadequate to cope with the demands of the community environment. As such, there is a growing need for purpose-built personal-use devices to meet the different challenges faced in these environments. To meet this, the creators of new personal-use devices should endeavor to restart the ideation and design process from the renewed base of user research and testing from the perspective of the home and community environment, to add to the knowledge retrieved from the design process in the clinical setting.

Directly testing a device in the home or community setting carries its own complexities, as there are many uncontrollable factors, including different environments. This can not only carry more risks for the user but also operational difficulties for the clinical personnel. Inpatient rehabilitation hospitals can be set up to provide a diverse environment with various surfaces, obstacles, and scenarios that mimic real-life situations, as well as simulated home environment spaces such as rehabilitation kitchens (Khan et al., 2019). Within these simulated settings, testing the feasibility for the user to carry out basic and advanced skills with the exoskeleton can occur in a controlled and supervised manner to ensure safety, and gain vital information for the design process (Khan et al., 2019). This information can provide the essential base for the design of a device that is suitable for use in the community setting following a user-centered approach (van Dijsseldonk et al., 2017; Spungen et al., 2013; Yang et al., 2015).

The ABLE Exoskeleton device has been developed for clinical use following a co-creation process to iterate its design (Porras-Martínez et al., 2023). The first study conducted in a hospital-based setting with the previous knee-powered prototype (*ABLEknee*) demonstrated that users were able to complete basic skills such as sit-to-stand, walk 10 m, turn 180 degrees, and stand-to-sit safely within a 12-session training program (Wright et al., 2023). Internal review of the quantitative data from this study, alongside qualitative user feedback from therapists and participants with SCI, was used to direct the ideation for design changes to the ABLE Exoskeleton to better adapt to the needs of different users. Design changes implemented from this process are reviewed in detail in a previous publication (Porras-Martínez et al., 2023). Main changes included improvements to the hardware of the device (i.e. redesigning the lumbar module and adding hip motors for better trunk stability), as well as the software (i.e. implementation of a turning function and a new mode to transfer into the exoskeleton). Testing of this updated version of the ABLE Exoskeleton (*ABLEhipknee*) was performed again in a clinical environment with a similar protocol to the first clinical trial (Porras-Martínez et al., 2023). The later study provided evidence suggesting that the new ABLE Exoskeleton (*ABLEhipknee*) is safe and can be used to perform basic skills in the clinical setting, while being superior in terms of performance and user satisfaction to the knee-only-powered *ABLEknee* prototype (Porras-Martínez et al., 2023).

Since the new ABLE Exoskeleton has only been successfully tested for performing basic tasks in the clinical environment, the ability to perform advanced skills needed for home and community use with the device needs to be evaluated similarly to a framework proposed by van Dijsseldonk et al. (2017) and crucial feedback from end-users with SCI and therapists needs to be obtained in order to redesign the ABLE Exoskeleton to be used for independent future use in the home and community environment. Additionally, since the new hip-knee-powered version has only been tested with a limited sample size of users with SCI in the clinical setting, further testing with more SCI individuals with a wider range of impairments is also necessary to support the findings of the pilot study. Therefore, the primary objective of this study was to test the ABLE Exoskeleton (*ABLEhipknee* version) in a simulated community environment as an interim evaluation toward home use, which involved assessing its feasibility with a focus on the level of assistance and usability with persons with SCI. The secondary objectives were to assess the gait and functional performance within the ABLE Exoskeleton, the rating of perceived exertion, psychosocial impact, quality of life (QoL) and general health in a sample of participants with broad neurological impairments as a result of SCI. Results will be used to support the development of ABLE Human Motion’s personal-use exoskeleton, by providing meaningful insights on the limitations of the current device reported by persons with SCI and therapists. This will ensure that new features can be implemented in the future personal-use device to be appropriately ready for home and community environments.

## Methods

**Figure 1:**
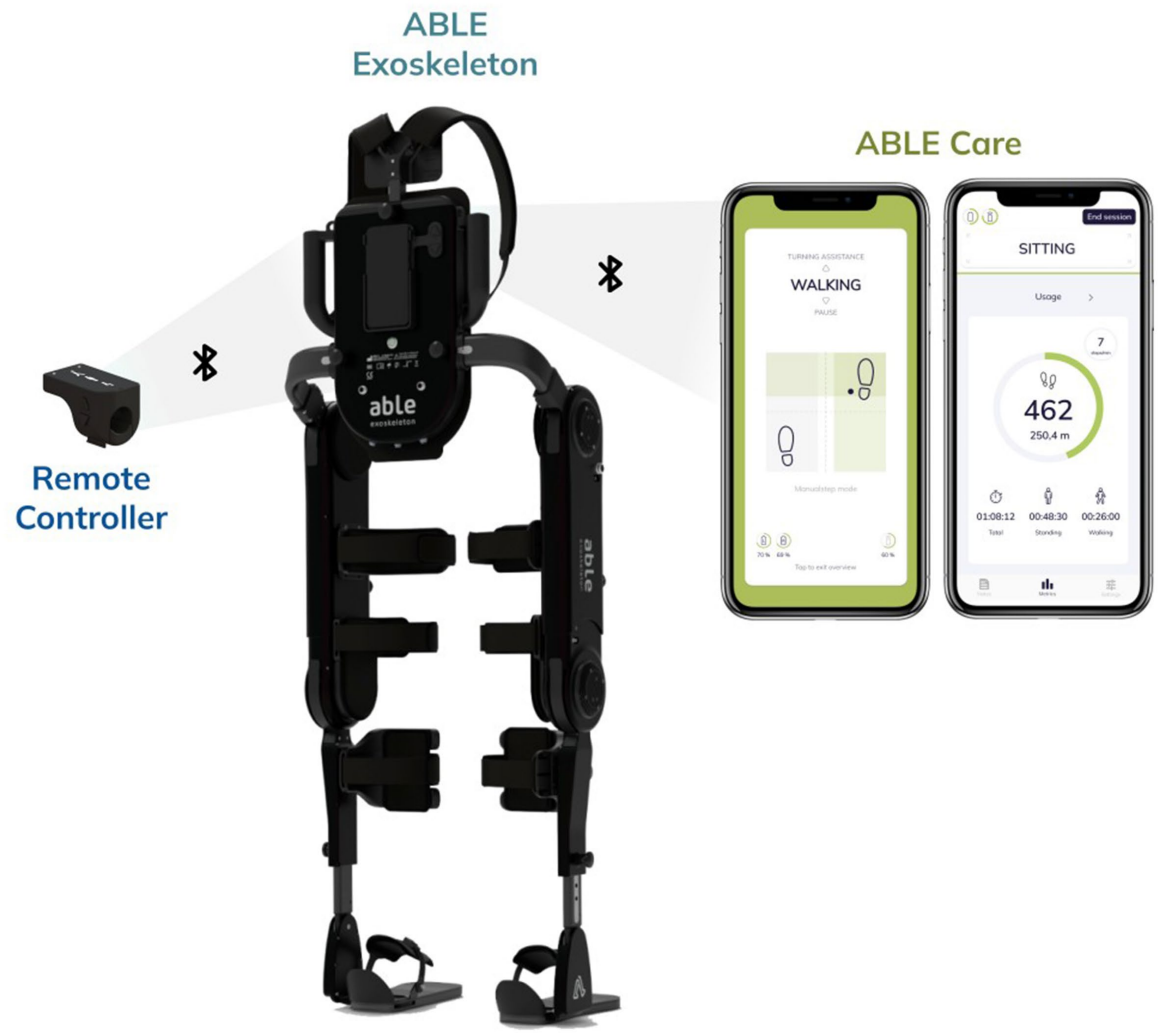
Overview of the ABLE Exoskeleton system. The ABLE Exoskeleton connects wirelessly to the ABLE Care app, which allows the therapist to configure and monitor the exoskeleton during supervised use, and to the Remote Controller, which allows the end user to operate the device autonomously.

The new hip-knee-powered ABLE Exoskeleton is a wearable, powered lower-limb robotic exoskeleton that actively assists individuals with mobility impairments to stand up, walk, turn, and sit down. The structure is a bilateral rigid frame that attaches to the user’s torso, legs, and feet through straps and rigid supports, with a total weight of 17 kg (Figure 1). It is composed of a lumbar module, two leg modules, two detachable foot modules, and an optional module with shoulder straps. Four battery-powered motors drive the knee and hip joints, assisting in flexion and extension. All other movement directions of the knee and hip joints are restricted. The ankle joints are articulated in plantarflexion-dorsiflexion using a spring mechanism.

The ABLE Exoskeleton is to be used with crutches or a walker for stability. It comes with an Android mobile phone with a pre-installed software application (ABLE Care), which communicates wirelessly with the device via Bluetooth and allows the therapist to configure and monitor the exoskeleton during a therapy session.

The exoskeleton is controlled using either the Therapist Controller (up and down buttons located on the lumbar module of the exoskeleton) or the Patient Controller (up and down buttons located on the Remote Controller attached to the walker or crutches). These controllers allow the users to transition between the different states of the exoskeleton: from sitting to standing, from standing to walking, from walking to turning, and vice versa. During walking, each step can be triggered by either the therapist or the end user. When done by the therapist, they hold the device using the handles and decide when to activate each step manually by pressing either of the pushbuttons (left for triggering the left hip and knee flexion-extension motion and right for the right one) that are located on the lumbar module of the device. When done by the end user, their intention to take a step is detected automatically using inertial measurement unit (IMU) sensors embedded in the exoskeleton. IMU sensors send motion data to the electronic control unit, which then analyzes the data and identifies the time instant to start a step cycle.

There are two automatic modes: Center of Mass, which triggers a step with the leg that is behind when the end user shifts their weight laterally and frontally, surpassing a predefined threshold; and Dynamic, which detects a forward motion of the pelvis measured through a change in the thigh angular velocity of the stance leg, to then trigger a step. This dynamic mode allows one to achieve a more dynamic and smoother gait pattern, by concatenating a step with the next one faster.

We conducted a prospective pretest-posttest quasi-experimental study in two European SCI centers (Institut Guttmann, Badalona, Spain and Heidelberg University Hospital, Heidelberg, Germany) from November 2022 until August 2023. The individual pre-post training period with 22 scheduled training sessions covered 5–8 weeks. The clinical trial was approved by the responsible local ethics committees and competent authorities of Spain and Germany, respectively (EUDAMED No.: CIV-20-07-034264). The study was conducted under the requirements of ISO 14155:2020 and European Regulation 2017/745 on medical devices (MDR).

Sampling was completed by the pre-screening of current and previous in-and outpatients at both sites. All patients who met the inclusion criteria and did not present with criteria for exclusion were asked to participate in the clinical trial. The inclusion and exclusion criteria of the study participants can be seen in Table 1. We excluded any individual with a history of lower-limb fragility fractures in the last 2 years (Bach Baunsgaard et al., 2018), and/or, who had five or more risk factors present for fragility fractures as cited by Craven et al. (2009). The International Standards for Neurological Classification of Spinal Cord Injury (ISNCSCI) and the Walking Index for Spinal Cord Injury (WISCI II) without the exoskeleton were evaluated at screening (and post-training).

**Table 1:** Inclusion and exclusion criteria.

Inclusion criteria	Exclusion criteria
18–70 years of age. Traumatic and non-traumatic SCI. Motor incomplete SCI with Neurological Level of Injury (NLI) C5-L5, or, motor complete SCI with NLI T1-L5. Time since onset of SCI > 6 months. Ability to give informed consent.	WISCI II without exoskeleton of >13. Five or more risk factors for fragility as stated by Craven et al. (2009). History of lower limb fragility fractures in the last 2 years. Deterioration >3 points of the total International Standards for Neurological Classification of Spinal Cord Injury (ISNCSCI) motor score within the last 4 weeks. Spinal instability. Modified Ashworth scale (MAS) of 4 in lower limbs. Unable to tolerate 30 min standing without clinical symptoms of orthostatic hypotension. Unable to perform a sit-to-stand transfer or stand in the device with assistance Psychological or cognitive issues that do not allow a participant to follow the study procedures. Any neurological condition other than SCI. Medically unstable. Severe comorbidities including any condition that a physician considers to not be appropriate to complete participation in the study. Ongoing skin issues. Height, width, weight or other anatomical constraints (such as leg length differences) incompatible with the device. Range of motion (ROM) restrictions in lower extremities that are incompatible with the device. Known pregnancy or breastfeeding.

In respect to the operation of the exoskeleton, all therapists conducting training sessions received 8 h of training for the exoskeleton device (4 h practical, 4 h theory) before the commencement of the study. A completed physiotherapy or occupational therapy education and more than 2 years of experience with the treatment of people with SCI were requirements for participation in the training. A total of 10 therapists were involved in conducting sessions across both sites, five of which had not previously used any version of the ABLE Exoskeleton, while the other five were involved in the first study with the *ABLEknee* prototype. None of the therapists involved had previously used the *ABLEhipknee* version. Due to varying availability of time for therapists due to other responsibilities, vacations or sick days it was not possible that completion of sessions was even across all therapists, however, it was ensured that all participants had at least one session with each therapist in their center.

**Figure 2:**
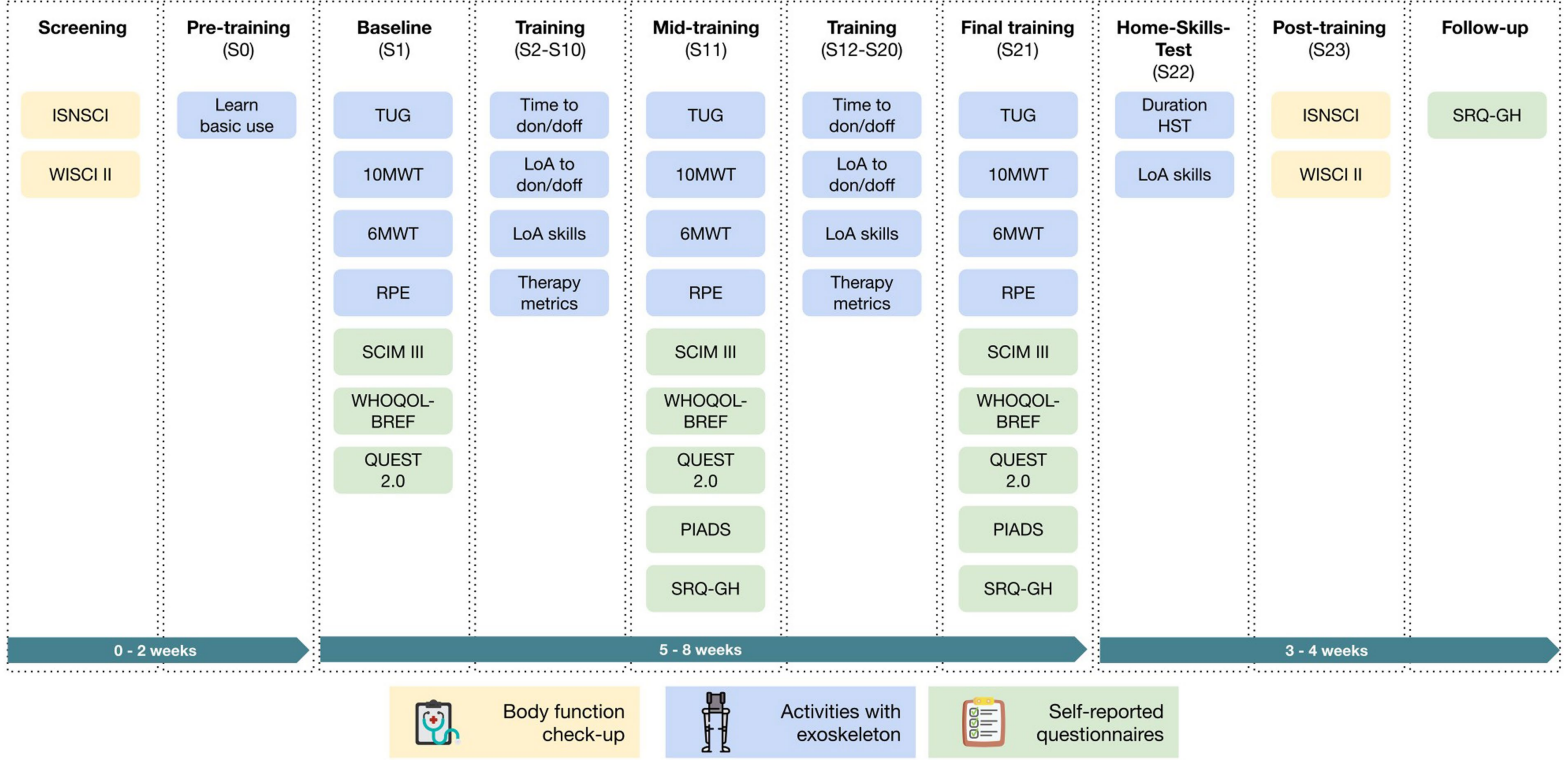
Overview of all study visits and measurements. Abbreviations are defined in the abbreviation section.

After screening for in-and exclusion criteria, a familiarization session with the exoskeleton was completed before commencing training. During this session, participants were educated on the operating mechanisms of the exoskeleton and guided through the basic use of the device: sit-to-stand, standing, weight shifting, stand-to-sit and walking, using crutches or frame and use of the Remote Controller as deemed appropriate for each individual. The walking aid and the step initiation mode was individually chosen according to the participant’s ability and may have changed as the study progressed. Participants then participated in a training program with the ABLE Exoskeleton three to five times a week until they had completed a total of 18 training sessions and four assessment sessions (Figure 2). The training sessions were scheduled for 60–90 min to include adjustments, donning and doffing, and data collection time. Therapy time (time spent standing, walking, or sitting in the exoskeleton) was intended to be at least 30 min per session. Each session was carried out by at least one trained therapist plus an additional therapist or assistant if required. During each training session, feasibility measurements were taken via evaluation of the performance of exoskeleton skills. The assessment sessions with the device were performed at different time points throughout training: Baseline (Session 1), Mid-training (Session 11), Final training (Session 21), and Home-Skills-Test (Session 22). A post-training assessment took place after the final training session, with a follow-up assessment conducted with participants 4 weeks later. At the end of the trial, therapists’ satisfaction with the device was evaluated. Participants were considered drop-outs if they did not complete a minimum of 10 training sessions and at least two of the three training assessment sessions (S1, S11, or S21).

Outcome measures were chosen according to the primary and secondary study objectives.

Adverse events (AE) were monitored and rated throughout the training program until follow-up by the Principal Investigator at each site. AE monitoring focused on skin changes, pain and any medical issue that required a medical review or change in medication.

The level of assistance (LoA) to don/doff the device was assessed every session by the therapists using the following grades: unable to perform, total, maximum, moderate, and minimal assistance, supervision, and independence as in the framework proposed by Wright et al. (2023) (see Supplementary material 1). Therapists were instructed to not assist the participant unless they requested help and/or there was a concern for safety if they were not assisted. The time to don/doff the device was also assessed every session using a standard stopwatch not linked to the device software.

Previous studies have varied in the exoskeleton skills that have been tested, with different evaluation methods executed depending on the judgment of the investigators (Spungen et al., 2013; Hartigan et al., 2015; Kozlowski et al., 2015). To bring more uniformity to this process and allow for the comparison of outcomes of different studies, a framework for measuring the progress in exoskeleton skills in people with complete SCI was developed by van Dijsseldonk et al. (2017). The proposed framework consisted of 27 exoskeleton skills tests, arranged into a hierarchy so that the difficulty increased with each tested skill. Due to space and facility restrictions across both centers, the full set of 27 skills from van Dijsseldonk et al. (2017) could not be conducted, therefore a revised set of 15 skills was attempted by participants over a training block of three consecutive sessions (sessions 2–4; 5–7; 8–10; 12–14; 15–17; 18–20), with the best LoA grade achieved for each skill in the training block recorded for the results. LoA was rated by the therapists using the same scale as in Wright et al. (2023) (see Supplementary material 1) with the same instructions regarding assisting the participant as for don/doff. The goal for skills was to be performed with only supervision or independently by the end of training block 6. The first six skills in Table 2 were categorized as basic skills, while skills 7–15 were categorized as advanced skills. The two skills *Arrest gait on command* and *Walk a 90° curve to the right/left* from the previous study with the knee-powered prototype of the ABLE Exoskeleton (*ABLEknee*) (Wright et al., 2023) were added to basic skills due to their position in the hierarchy established by van Dijsseldonk et al. (2017). Participants were instructed to use any technique within the exoskeleton that allowed them to accomplish the skill, i.e., during turning, participants were free to decide if they wished to use the turning assistance feature of the exoskeleton or remain in safe standing or walking mode to make steps round to complete a turn. Skills that were to be attempted in each session were set up in rehabilitation spaces at each center to form the simulated home and community environments for the study. A full list of the skills can be seen in Table 2.

**Table 2:** Skills assessed during each training block of study.

Basic skills	1. Sit-to-Stand
	2. Stand-to-sit
	3. Walk 10 m
	4. Arrest gait on command
	5. Walk a 90° curve to the right/left
	6. Walk a 180° curve to the right/left (radius 1.8 m)
Advanced skills	7. Arrest gait nearby a box or table and move a cone at chest height
	8. Take out phone from pocket and send a message
	9. Pass a narrow passage (e.g., door)
	10. Arrest nearby a door, open the door away from you/toward you and enter
	11. Arrest near a chair, pivot turn and sit down
	12. Walk on an upward/downward slope
	13. Walk over a martial arts mat (185*60 cm, height 1.5 cm)
	14. Walk a slalom around four poles (distance between poles 3.0 m)
	15. Enter an elevator, ride to another floor, and exit without requiring the elevator door to be held open

The 15 skills were categorized into six basic skills and nine advanced skills.

**Figure 3:**
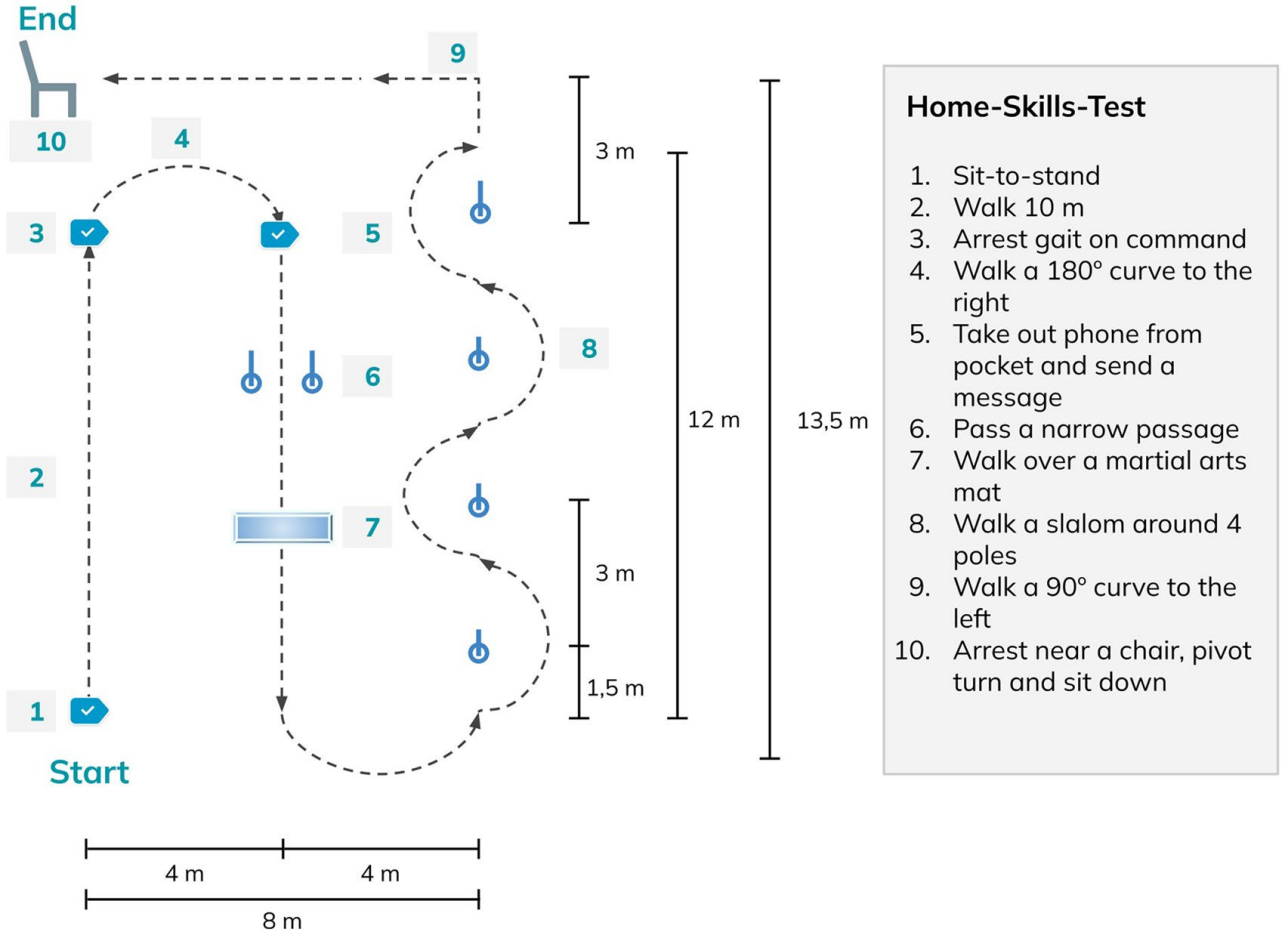
Home-Skills-Test conducted with the exoskeleton during session 22.

A Home-Skills-Test (HST) was performed in session 22 and consisted of 10 skills for home and community environments, set out in a continuous sequence to simulate daily life situations (see Figure 3). The HST is an adaptation of the Final Skills Test performed by van Dijsseldonk et al. (2017) to allow the test to be performed identically in both centers. For all skills of the HST, the LoA to complete each task was rated by the therapists using the same scale as for skill completion in training sessions (see Supplementary material 1), with the same instructions regarding assisting the participant. The time taken to complete the test was also documented.

The study participants’ ability to use the device was also assessed by the usage metrics recorded by the device: standing time, walking time, distance walked, and number of steps.

Gait performance and the rating of perceived exertion (RPE) were assessed by conducting standardized assessments with the ABLE Exoskeleton at sessions 1, 11, and 21: Timed Up and Go (TUG), 10-Meter Walking Test (10MWT), 6-Minute Walking Test (6MWT), and the Borg-RPE scale (BORG). Standardized rest breaks of 10 min were included between walking tests to ensure the results of each test were reliable and valid. Timing for all walking tests was taken using a standard stopwatch not linked to the device software, with participants walking the relevant distances for the TUG and 10MWT on pre-marked out courses marked out on the floor. For the 6MWT, a course of 50 m was marked out, with participants turning 180 degrees once they reached the end of the track.

The assessments explained previously cover the function and activity domains of the International Classification of Functioning, Disability and Health (ICF) (International Classification of Functioning, 2001), therefore, to consider the personal and environmental domains of the ICF, we placed a special emphasis on the inclusion of patient-reported outcome measures regarding the exoskeleton use. The Quebec User Evaluation of Satisfaction with assistive Technology (QUEST 2.0), the Psychosocial Impact of Assistive Devices Scale (PIADS), the WHO Quality of Life-BREF (WHOQOL-BREF), and a Self-Report Questionnaire on the perceived impact of the use of the device on General Health (SRQ-GH) were all completed by participants. While QUEST 2.0 (Kozlowski et al., 2015; Fernández-Vázquez et al., 2021; Puyuelo-Quintana et al., 2020), PIADS (Fundarò et al., 2018), and WHOQOL-BREF (Salvador-De La Barrera et al., 2018) have been previously used in clinical studies to measure satisfaction, psychological impact, and QoL, respectively, the questionnaire SRQ-GH was designed by the authors to identify participants perceived changes in their general health during and after training with the ABLE Exoskeleton. The SRQ-GH includes seven categories: Cardiovascular health, Musculoskeletal pain, Neuropathic pain, Bladder and bowel, Skin, Spasticity and Sleep quality. Participants rated each category on a seven-point Likert scale for perceived changes over the last 15 days, whereby 0 represents no change at all, +1, +2, +3 represents a mild, moderate or maximum positive change, respectively, and −1, −2, −3 represents a mild, moderate or maximum negative change, respectively. If a change is reported, participants are asked if they perceive this change to be due to exoskeleton training (Supplementary material 2).

Therapists’ satisfaction was measured with the QUEST 2.0 at the end of the trial once all participants had completed the follow-up. Following the end of the trial, general feedback was sought from therapists who participated in the trial.

During the training period, participants performed each skill at least once per block of three consecutive sessions. Therefore, not all the skills were attempted in every session, and not all the skills were performed the same number of times by all participants. To account for this variability, the analysis of the LoA for each participant was done using the best LoA grade recorded per task within a block.

Similarly, the analysis of device usage metrics for each participant was performed using the best session recorded within a block, since there were sessions where participants spent more time practicing skills that required them to focus on standing balance or other abilities rather than just walking. To determine the best session record, we considered the session with the highest number of steps as the main selection criterion. In the case of ties in the number of steps between sessions, the second criterion used was the longest time spent walking. For each participant, all outcomes of the device usage metrics analyzed were the ones recorded in the session selected.

Quantitative variables were summarized using standard descriptive statistics [median, interquartile range (IQR), mean, standard deviation (SD), and minimum and maximum]. Qualitative variables were described using group sizes and frequencies. The differences between pre-post training outcome measures were analyzed using the non-parametric Wilcoxon test. For multiple measures, the Friedman test and Wilcoxon *post-hoc* test with adjusted Bonferroni-Holm correction were calculated using the statistical software R (v4.2.1). Values of *p* ≤ 0.05 were considered statistically significant.

## Results

Ten individuals with SCI participated in the study. Five of the participants had motor complete injuries (AIS grade A or B) with NLI between C8 and T9, while the other five had motor incomplete injuries (AIS grade C or D) with NLI from C5 to T11. Seven of the participants were in the chronic stage of SCI (>1-year post-injury), three were in the sub-acute stage (3–12 months post-injury), and the time since injury to recruitment in the study ranged from 6 months to 19 years. On average, participants were 44.4 ± 24 years old and mostly male (70%).

**Figure 4:**
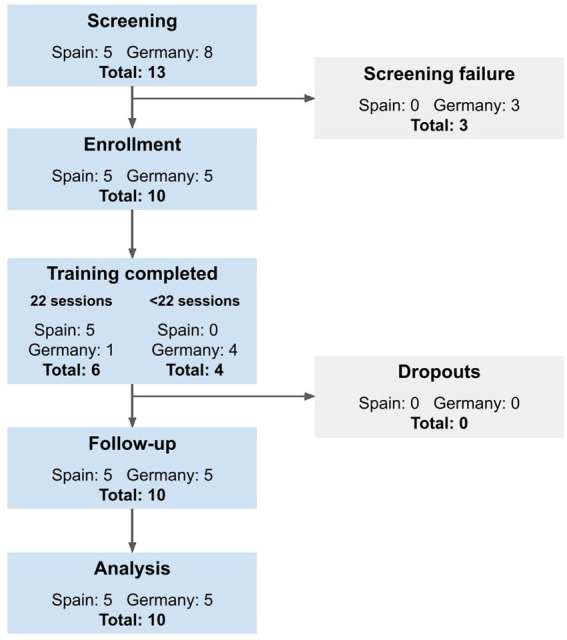
Study flow chart of recruited participants in line with the STROBE statement (http://www.strobestatement.org).

On average, 17.00 ± 1.70 training sessions were completed per participant. Six participants (60%) completed all 18 training sessions, two participants missed one session, one missed three sessions and one missed five sessions. Missed sessions were due to health reasons unrelated to training, along with the required maintenance periods of the device. Nine participants (90%) completed all four assessment sessions, one completed one out of three clinical gait measures in the baseline assessment and one missed the HST. All participants remained in the study until the follow-up visit, thus there were no drop-outs (see Figure 4).

A total of 27 AEs were reported during the 209 sessions (training: *n* = 170, assessment: *n* = 39) performed throughout the study across the two sites. Six (22.2%) of these AEs were classified as device-related, i.e., rated as “related” or “probably related” to the device by the assessors (four skin markings, one increase in neuropathic pain, one bruise on a therapist’s hand). None of the AEs were rated as serious, and there were no falls.

Donning and doffing of the device required an average total time of 10 min and 23 s (± 3:30 min:s) across the whole training period. Mean total time taken for donning and doffing significantly decreased (*p* < 0.05) from 11 min and 50 s in session 1 (S1), to 9 min and 2 s in session 21 (S21) (Supplementary material 3). No statistically significant correlation was found between the time to don/doff the device and time post-injury, AIS, or NLI.

**Figure 5:**
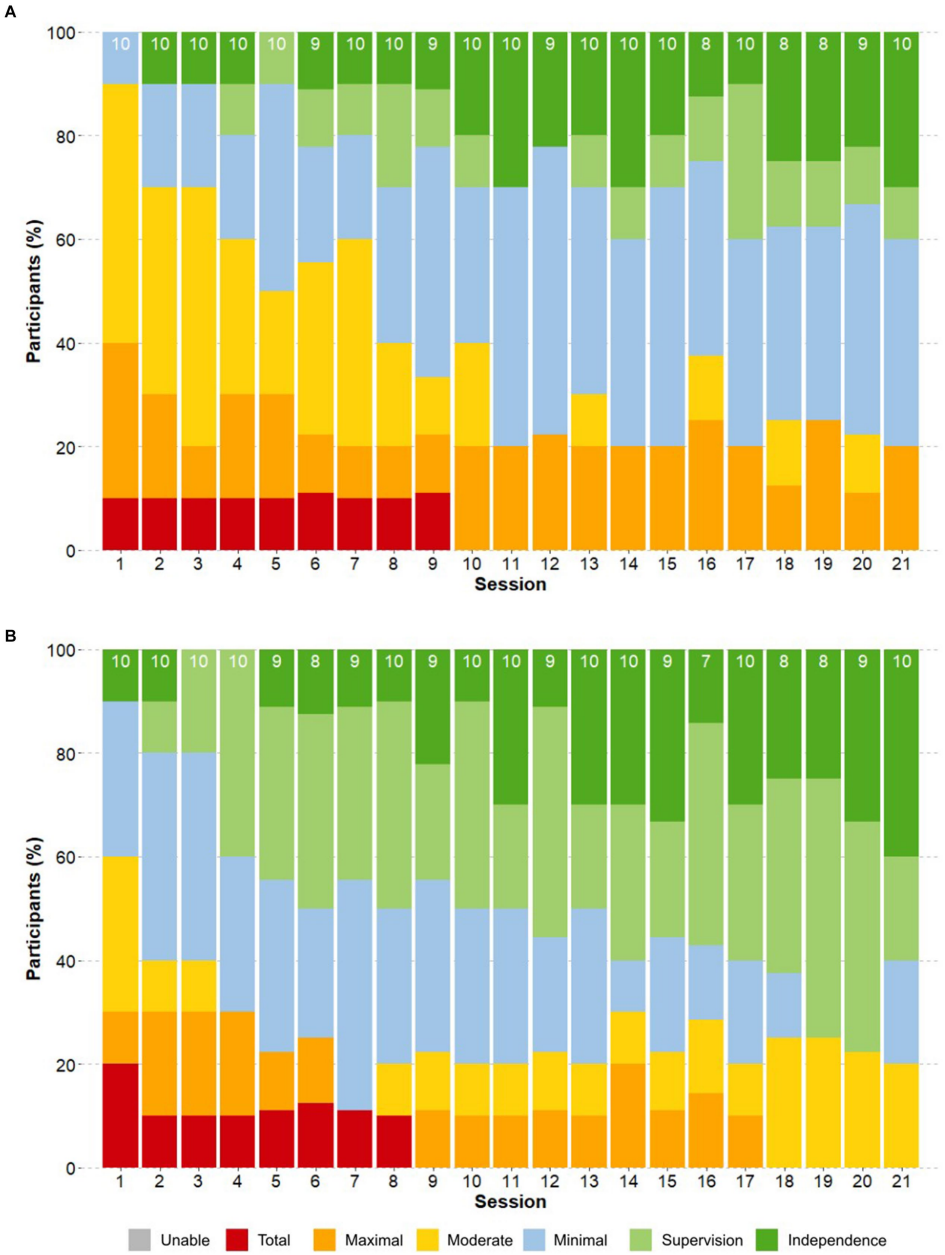
Progression of the Level of Assistance in donning and doffing. The proportion of participants that required a certain level of assistance (LoA) for **(A)** donning and **(B)** doffing is shown throughout the training sessions. The number on top of each bar indicates the total number of participants who attempted the task in that session.

At the end of the training, the majority of participants (80%) were able to complete donning either independently (30%), with supervision (10%), or with minimal assistance (40%) (see Figure 5A). The same 80% of participants were able to complete doffing either independently (40%), with supervision (20%), or with minimal assistance (20%) (see Figure 5B). The two participants with C6 AIS C lesions in the sub-acute phase required maximal assistance to don, and moderate assistance to doff the device at the end of training. A statistically significant (*p* < 0.05) negative correlation was identified between the phase after SCI and the LoA to don/doff. No statistically significant correlation was found between LoA to don/doff and AIS or NLI.

**Figure 6:**
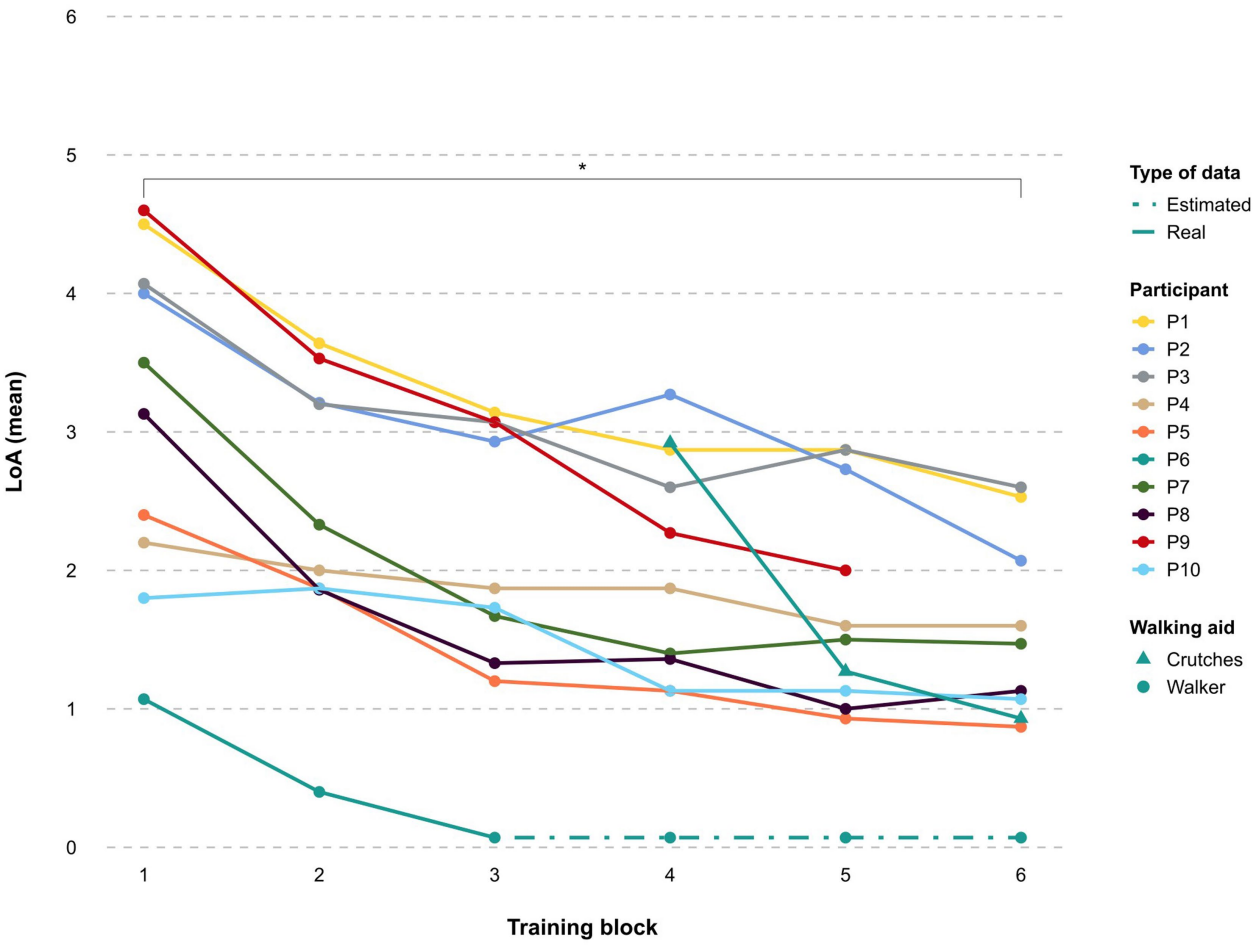
Learning curve. The mean level of assistance (LoA) per participant to achieve all 15 skills during the training period is shown throughout the training blocks. 6: unable to perform, 5: total assistance, 4: maximum assistance, 3: moderate assistance, 2: minimum assistance, 1: supervision, 0: independence.

Independence to carry out all 15 skills in the device increased significantly for all participants as the training progressed (*p* < 0.05) (see Figure 6). While in the first session, steps were equally initiated manually by therapists and automatically by study participants, the use of the automatic step initiation increased progressively over the sessions, reaching a percentage of over 95% from S6 and 100% from S18 onwards for all participants. The three participants in the sub-acute stage required higher LoA and demonstrated a slower learning curve than participants in the chronic phase throughout the study regardless of NLI or AIS grade. A statistically significant (*p* < 0.01) negative correlation was identified between the phase after SCI and LoA for skills completion. No statistically significant correlation was found between LoA for skill completion and AIS or NLI.

**Figure 7:**
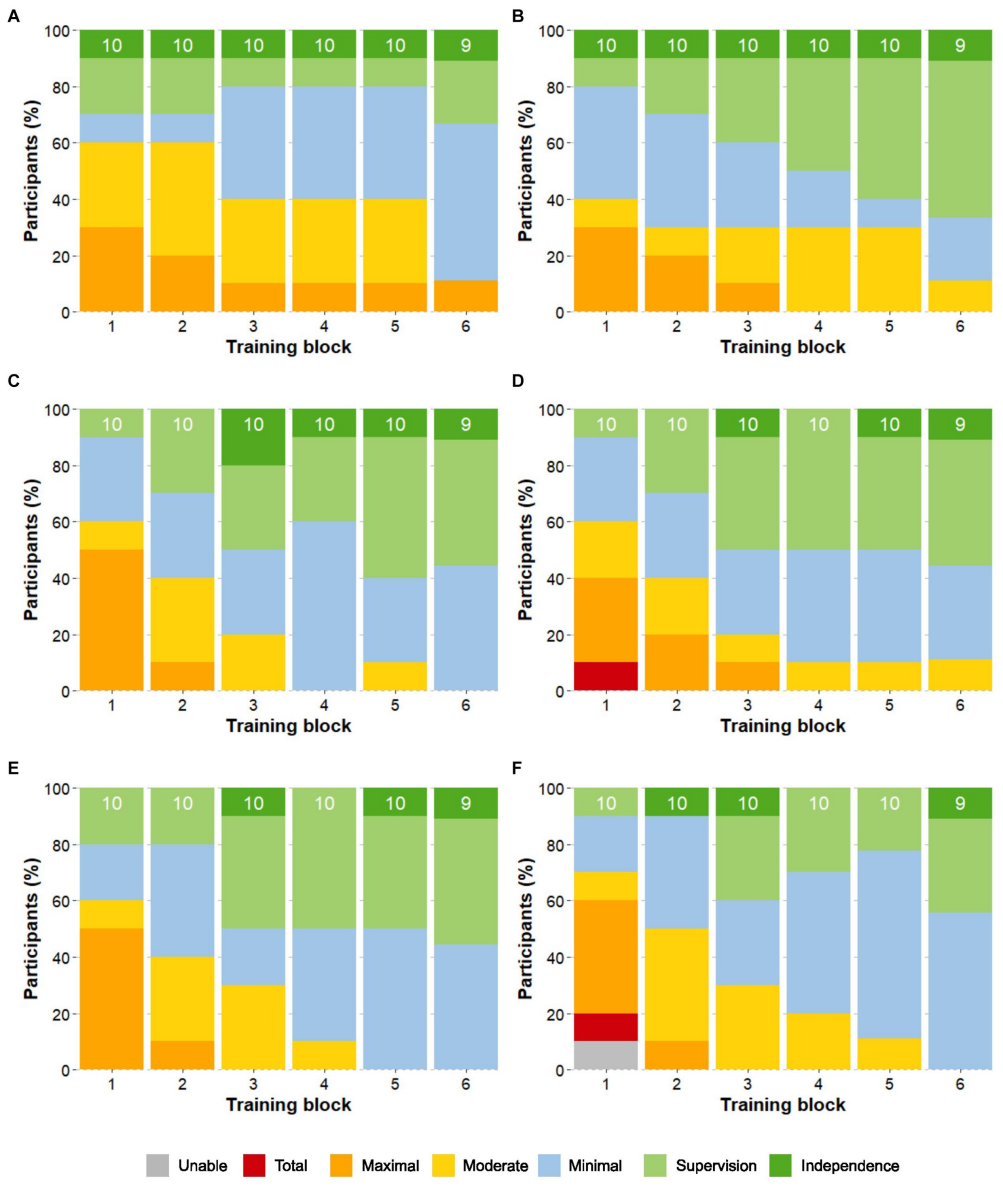
Progression of the Level of Assistance of the basic skills. Skills are: **(A)** Sit-to-Stand **(B)** Stand-to-sit **(C)** Walk 10m **(D)** Arrest gait on command **(E)** Walk a 90° curve **(F)** Walk a 180° curve. The proportion of participants that required a certain level of assistance (LoA) for each of the basic skills is shown over the course of the training blocks. The number on top of each bar indicates the total number of participants that attempted the skill.

The LoA required to complete the basic skills decreased from beginning to end of the study (see Figure 7). By training block 6, at least 50% of participants were able to complete the skills *Stand-to-sit, Walk 10m, Arrest gait on command,* and *Walk a 90 degree curve* only with supervision or less. The skill *Sit-to-stand* was achieved with supervision or less by 33% of participants, while *Walking a 180 degrees curve* with supervision or less was achieved by 45% of participants. One participant required maximal assistance for *Sit-to-stand* by block 6, while the other eight participants performed this skill with minimal assistance (*n* = 5), supervision (*n* = 2), or independently (*n* = 1).

**Figure 8:**
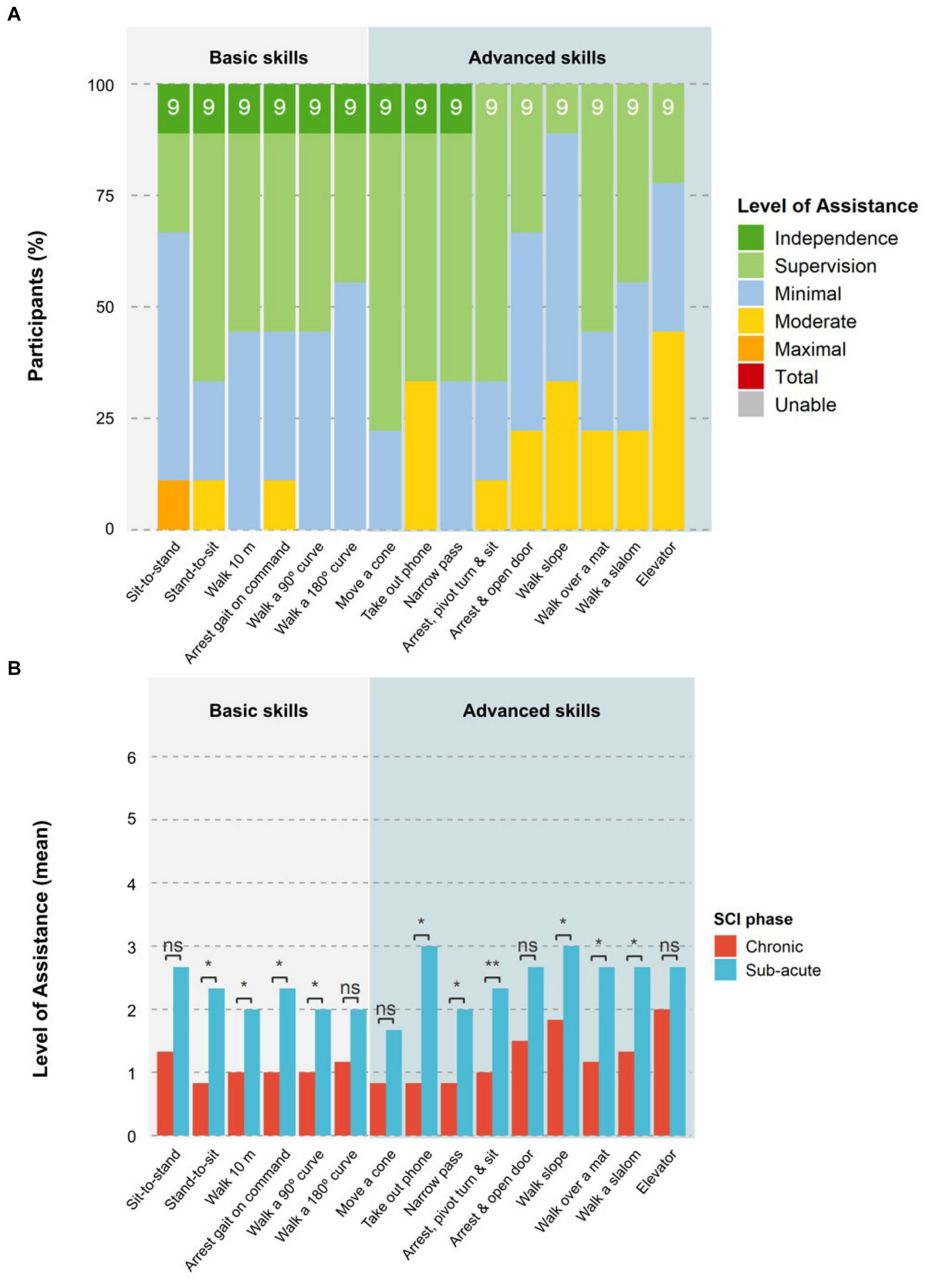
Level of assistance per task at the end of training. **(A)** The proportion of participants that required a certain LoA for each task at the last block of the training period (block 6) is shown. The number on top of each bar indicates the total number of participants that attempted the skill. **(B)** The mean LoA of participants in the chronic or sub-acute phase for each task at the last block of the training period (block 6) is shown. Statistical significance is shown as ns = not significant, *p < 0.05, and **p < 0.01.

Results at the end of the training block 6 showed that the advanced skills of *Arrest gait nearby a box or table and move a cone at chest height, Take out phone from pocket and send a message, Passing a narrow passage, Arrest nearby a door, Open the door away from you/toward you and enter,* and *Walk over a martial arts mat* were performed with only supervision or less by at least 50% of participants. Up to 45% of participants were able to *Walk a slalom around 4 poles* with only supervision by block 6, while 33% could *Arrest near a chair, pivot turn and sit down* with supervision. *Walking on an upward/downward slope* and *Enter an elevator, ride to another floor, and exit without requiring the elevator door to be held open* were the most complex tasks, with 33 and 45% of participants, respectively, requiring moderate assistance to complete the tasks by block 6 (Figure 8A).

Participants in the chronic phase performed advanced skills consistently better than those in the sub-acute phase, while NLI and AIS had no significant impact. The chronic group required a significantly (*p* < 0.05) lower LoA for six of the nine advanced skills at block 6 (see Figure 8B). The three advanced skills that showed no significant differences between the groups were: *moving a cone at chest height, pivot turn and sit down*, and *entering an elevator*.

**Figure 9:**
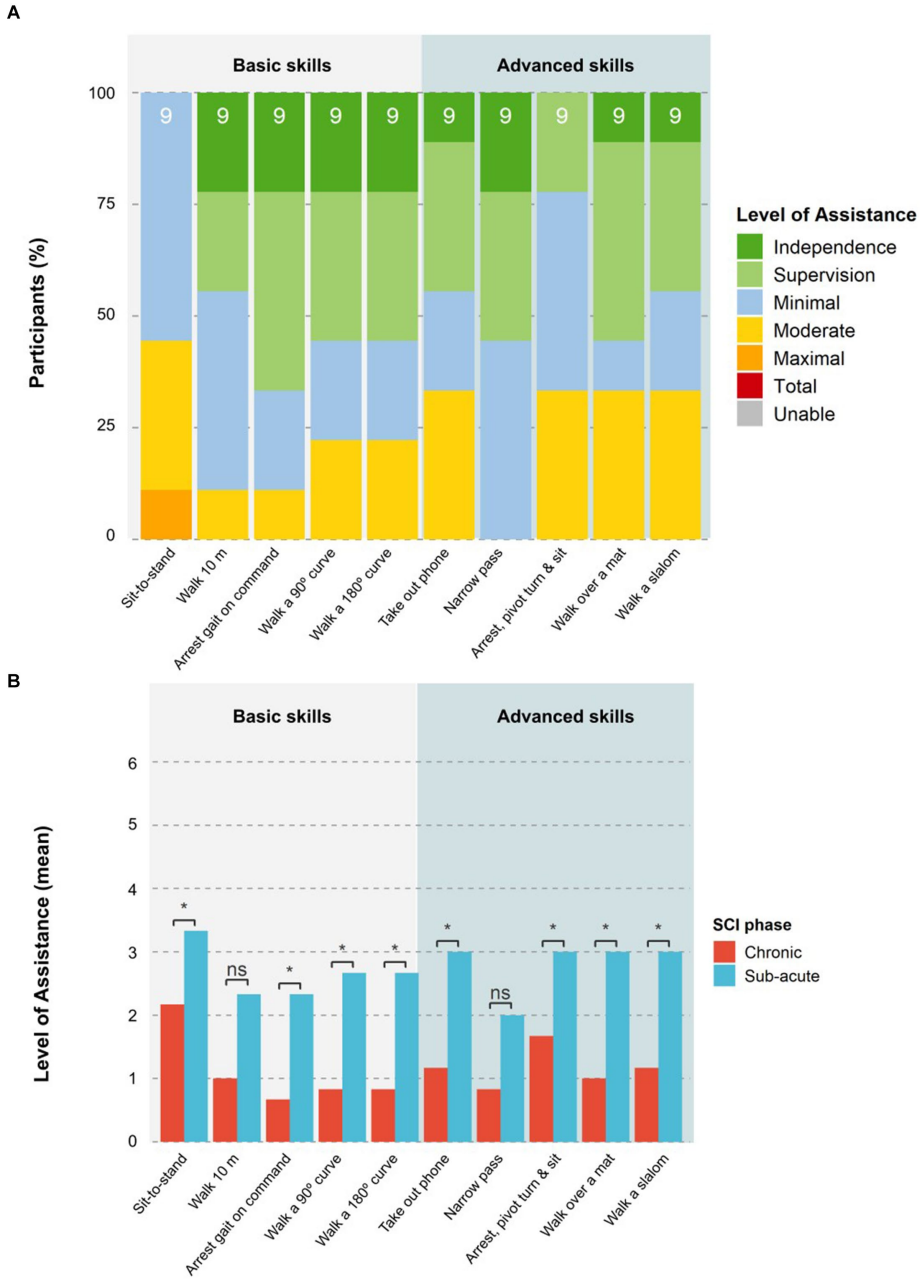
Level of assistance per task in the Home-Skills-Test. **(A)** The proportion of participants that required a certain LoA for each task in the HST is shown. The number on top of each bar indicates the total number of participants that attempted the task. **(B)** The mean LoA of the participants in the chronic and sub-acute phase for each task performed in the HST is shown. Statistical significance is shown as ns = not significant, *p < 0.05, **p < 0.01.

Nine of the 10 participants attempted the HST at session 22. The median duration to complete the test was 7:45 min:s (IQR 5:09; mean 10:34 ± 7:48). One participant needed 29:44 min:s to complete the HST due to increased spasticity, resulting in a slower walking speed and breaks that were required by the device to cool down the hip motors. Five participants also had issues with the speed of transition between exoskeleton states (i.e., for turning and walking) that led to delays in the performance of skills that required a different exoskeleton state than the previous skill in the sequence. Half of the skills (*Arrest gait, Walk a 180 degrees curve, Walk a narrow passage, Walk over a martial arts mat,* and *Walk a 90 degrees curve*) were performed by at least 50% of participants with only supervision or better. All skills apart from *Sit-to-stand* and *Pivot turn to sit down* were performed independently by at least one participant. The basic skill *Sit-to-stand* remained the most challenging task for all participants, with one participant in the sub-acute phase requiring maximal assistance. Participants in the chronic stage required a significantly (*p* < 0.05) lower LoA for eight of the 10 skills in the HST (Figure 9) than those in the sub-acute phase. The skills *Walk 10m* and *pass a narrow passage* were the only two skills that showed a non-significant difference between chronic and sub-acute phase participants. NLI and AIS showed no significant impact on HST results for LoA or time taken.

A mean active therapy time (time spent upright in exoskeleton) per session of 31.83 ± 8.45 min (median: 31 min, IQR: 9 min) was achieved, with 62.8% of this time spent walking. There were no significant differences in the walking time between beginning and end of study (for an overview of the assessment schedule see Supplementary material 3). An average of 454.64 ± 283.40 steps were taken per session, with a significant increase (*p* < 0.05) in the number of steps between beginning (323.00 ± 186.68 steps) and end of study (563.22 ± 301.13 steps) (for data table of assessments, see Supplementary material 3). An average of 162.91 ± 113.61 m was walked per session, with a significant increase (*p* < 0.05) in the distance walked per session between beginning (108.96 ± 64.88 m) and end of study (198.17 ± 123.23 m).

Significant improvement (*p* < 0.05) was found in the 10MWT with the ABLE Exoskeleton between S1 (0.12 ± 0.06 m/s) and S21 (0.17 ± 0.06 m/s) and in the 6MWT between S1 (45.38 ± 16.71 m) and S21 (58.25 ± 26.84 m). Two participants (participants 02 and 03 in Table 3) had a reduction in distance for the 6MWT and slower 10MWT time at S21 compared to S11 due to spasticity and spasms, resulting in a slower walking speed and breaks that were required by the device to cool down the hip motors during these tests. No significant difference was detected for the TUG or BORG on Wilcoxon signed rank tests between the start and end of the study, however, six participants had intermittent issues with the speed of transition between exoskeleton states (i.e., for turning and walking) that led to delays in the turning aspects of the TUG. At the baseline assessment (S1), 53.26% of steps across all participants were initiated by the therapist in manual mode. In contrast, for those who completed the walking tests at the Mid-training (S11), Final training (S21), and HST (S22) sessions, all steps were initiated by the participants in automatic mode.

**Table 3:** Overview of the characteristics of each study participant.

Participant	Sex	Age	SCI phase at day of IC	NLI	AIS	LEMS	WISCI II	Max. MAS hip flex-ext*	Max. MAS knee flex-ext**
1	M	31–40	Sub-acute	C5	C	16	1	0	2
2	M	41–50	Sub-acute	T4	B	0	3	3	3
3	M	21–30	Sub-acute	C6	C	13	0	2	3
4	F	21–30	Chronic	C8	B	0	9	0	1+
5	M	61–70	Chronic	C5	D	26	7	0	0
6	F	31–40	Chronic	T11	C	7	9	0	0
7	M	51–60	Chronic	C5	D	21	6	1+	2
8	M	51–60	Chronic	T6	A	0	0	1	2
9	F	41–50	Chronic	T9	A	0	0	3	3
10	M	31–40	Chronic	T8	A	0	0	1	1+

Ranges of age are given to ensure the anonymity of study participants. IC, Informed consent; NLI, Neurological level of injury; AIS, American Spinal Injury Association (ASIA) Impairment Scale; LEMS, Lower extremity motor score; WISCI II, Walking index for spinal cord injury II without exoskeleton; MAS, Modified Ashworth scale. *Max. MAS hip flex-ext shows highest score on MAS in right/left hip for flexion or extension at screening. **Max. MAS knee flex-ext shows highest score on MAS in right/left knee for flexion or extension at screening.

**Figure 10:**
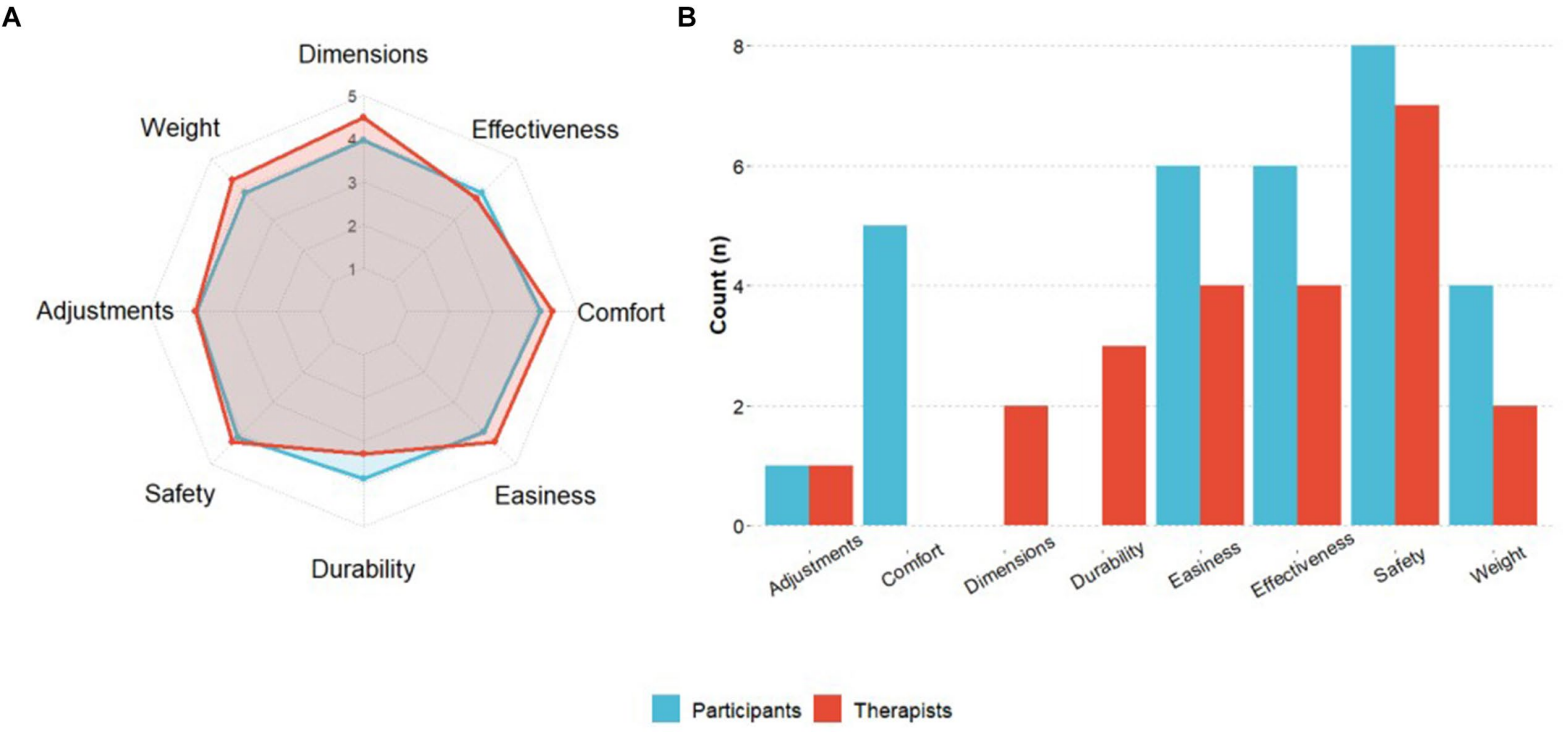
QUEST 2.0 results. **(A)** The participants’ satisfaction with the device at the end of training and the therapists’ satisfaction post-study completion. Blue and red dots represent the mean scores for study participants and therapists per QUEST 2.0 item. **(B)** The figure shows the number of participants and therapists who selected each item as the most important.

The mean satisfaction with the exoskeleton was rated by participants (total QUEST 2.0 score) as 29.60 ± 7.40 out of 40 (median: 29.50, IQR: 10.20) at S21. No significant differences were found between visits when the QUEST 2.0 was performed (S1, S11, S21). Results for each rated item are shown below in Figure 10A. “Safety” (mean 3.90 ± 0.99; median: 4.00, IQR: 1.50) and “Comfort” (mean 3.90 ± 1.10; median: 4.00, IQR: 2.00) were the best-rated items, followed by “Ease of use” (mean 3.70 ± 0.82; median: 4.00, IQR: 0.75). “Safety” was also rated as the most important category for the majority of the participants (80.0%), followed by “Effectiveness” (60.0%) and “Ease of use” (60.0%) (see Figure 10B).

A total of eight therapists completed the QUEST 2.0, scoring a total average satisfaction of 30.90 ± 4.64 (median: 31.50, IQR: 2.25) out of 40. The “Dimensions” of the device was the best-rated category (mean 4.38 ± 1.06; median: 5.00, IQR: 1.00), followed by “Comfort” (mean 4.25 ± 0.71; median: 4.00, IQR: 1.00), “Weight” (mean 4.12 ± 1.13; median: 4.50, IQR: 1.25) and “Ease of use” (mean 4.12 ± 0.64; median: 4.00, IQR: 0.25) (see Figure 10A). “Safety” was also rated as the most important category by therapists (87.5%), followed by “Effectiveness” (50.0%) and “Ease of use” (50.0%) (see Figure 10B).

User feedback to further enhance the development of the personal home-use exoskeleton was sought from participants and therapists. Therapists who regularly used the device highlighted the need to manage the overheating of the motors when used with participants with high levels of spasticity if the device was to be used in a home environment. Further feedback received from therapists also highlighted participants had more difficulty with the sit-to-stand sequence, a slowness in transitions between exoskeleton states (i.e., turning to walking states) which affected tasks such as entering the elevator, while end users identified the need for a richer display on the Remote Controller to have more clarity about what exoskeleton state the device was in.

**Figure 11:**
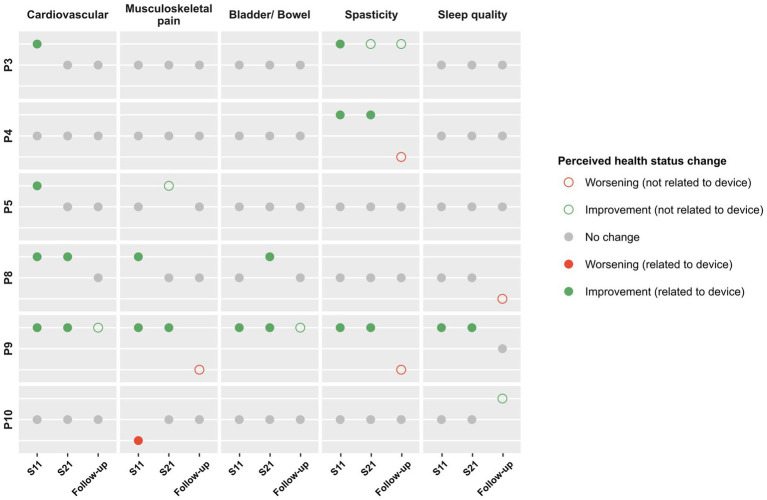
SRQ-GH results. The responses to the SRQ-GH among participants who reported a perceived change in health status due to the device at mid-training (S11), final training (S21) and follow-up, by category.

The results of the SRQ-GH demonstrated perceived improvement due to the device at mid or final training assessments for cardiovascular fitness (four participants), musculoskeletal pain (two patients), bladder/bowel (two participants), spasticity (three participants), and sleep quality (one participant). At follow-up, one participant reported a worsening not related to the device for musculoskeletal pain and spasticity and another participant reported worsening not related to the device for sleep quality. However, both had not previously reported any impact at mid or final training. One participant reported worsening of musculoskeletal pain due to the device at mid-training assessment, which did not change in the later assessments. No participants reported worsening due to the device for the other categories in any of the assessments (Figure 11).

Quality of life as measured by the score of items regarding the general perception of QoL and general health in the WHOQOL-BREF showed no significant change from baseline to final training session (Supplementary material 3). Overall psychosocial impact measured by the PIADS score at the end of training was positive (mean 18.90 ± 16.07, median: 17.50, IQR: 18.00) as well as in the three subscales: “Adaptability” (mean 7.10 ± 5.53, median: 6.00, IQR: 7.00), “Competence” (mean 6.80 ± 7.05, median: 4.50, IQR: 8.25), and “Self-esteem” (mean 5.00 ± 4.50, median: 5.00, IQR: 5.50). There was no significant difference between mid-training and final training scores (Supplementary material 3).

## Discussion

The knee-only-powered ABLE Exoskeleton prototype was designed and previously tested for clinical use, therefore this present study aimed to test the feasibility and usability of the new hip-knee-powered ABLE Exoskeleton (*ABLEhipknee*) to perform skills for home and community use, with the aim to support the user-centered design process of a new personal-use exoskeleton that ABLE Human Motion aims to create.

A distinct improvement in the mean rate of AEs per study participant and session was seen in this current study (*n* = 26; AE rate 0.13 per participant and session), compared to the study with the previous *ABLEknee* prototype (*n* = 81; AE rate 0.45 per participant and session) across a similar number of training sessions (209 vs. 242 sessions) (Wright et al., 2023). Importantly, none of the AEs in either study were serious, with no falls despite the training of more complex tasks in the current study. Skin marking and pain were the main issues encountered by users in this study, which have been noted as common AEs of use in studies with similar devices (Spungen et al., 2013; McIntosh et al., 2020; Tefertiller et al., 2018).

Completing the donning and doffing of an exoskeleton can be a complex task for persons with SCI, with the achievement of independence often impeded by the motor control available to the user. A previous study with the Indego exoskeleton found that no user with tetraplegia (C5-C8 injury level) could don/doff the device independently due to reduced hand function, while only one out of five participants with upper paraplegia (T1-T8 injury level) achieved independence (Hartigan et al., 2015). Similarly, in the trial with the *ABLEknee* prototype, one participant with tetraplegia required moderate assistance with donning and doffing by the end of the training, while the majority of other participants (68.8%) with paraplegia were able to complete both donning and doffing either independently (25%), with supervision (18.8%) or with minimal assistance (25%) (Wright et al., 2023). In this current study, the only two participants who required more than minimal assistance had a cervical SCI in the sub-acute phase. A significant improvement from the results found with the *ABLEknee* prototype was seen in three participants with tetraplegia or high-thoracic paraplegia in the chronic phase who were able to don and doff the device independently, while the majority of the rest of the participants still achieved donning and doffing with minimal assistance or supervision only. An average total donning and doffing time of 10 min and 23 s, with low levels of assistance, is favorable when compared to a previous study with the Indego exoskeleton that demonstrated an average total donning and doffing time of 11 min and 45 s (Tefertiller et al., 2018). The previous study with the *ABLEknee* prototype presented an average total time for donning and doffing of the device of 6 min and 50 s (Wright et al., 2023). One reason for this could be the modified design of the *ABLEhipknee*, which required users to close more straps compared to the knee-powered prototype. However, it should be noted that 16 out of 17 (94.1%) participants in that previous study had NLI between T1 and L3, with maintained hand function and higher levels of trunk control than in this current study.

As can be seen in the learning curve (Figure 6), all participants using the ABLE Exoskeleton showed an improvement in the amount of assistance required to perform basic and advanced skills from the training block 1 to 6. Achievement of key basic skills like *Stand-to-sit, Walk 10m, Arrest gait on command,* and *Walk a 90 degree curve* with supervision or less assistance was higher in comparison to a previous study with the Indego exoskeleton, whereby the majority of the participants continued to require supervision or minimal assistance for walking 10 m by the end of training (Tefertiller et al., 2018). As acknowledged by Tefertiller et al. (2018), this may be in part due to the therapists’ lack of experience with the device, given that none of the therapists had experience with the new ABLE Exoskeleton (*ABLEhipknee*) prior to starting this study. Despite the more rostral average NLI among participants in this current study, achievement of the supervision only or less threshold was higher than in the study with the *ABLEknee* prototype for the skills of *Walk 10m* (18.8%) and *Stand-to-sit* (37.5%) (Wright et al., 2023). Although Sit-to-stand was the most challenging basic skill for all participants in this current study, it is worth noting that no participants in the study with the *ABLEknee* were able to achieve *Sit-to-stand* independently or with supervision (Wright et al., 2023). The skill of *Sit-to-stand* has been reported as a difficult task in other studies using the EKSO GT exoskeleton, which reported participants still required moderate to maximal assistance at study end after 18 training sessions (Gagnon et al., 2018). However, this skill represents a fundamental and essential aspect that should be addressed for future exoskeleton models to allow for independent use in the home and community setting.

Achievement of the supervision or less threshold in the advanced skills of *Arrest gait nearby a box or table and move a cone at chest height, Take out phone from pocket and send a message, Pass a narrow passage, Arrest nearby a door, open the door away from you/toward you and enter,* and *Walk over a martial arts mat* were met in half of the participants. As seen in previous studies, participants varied in their method of accomplishing these tasks, with some achieving the task of moving a cone by placing their hands on the table for support, while others leaned their body forward into the table to gain balance (Khan et al., 2019). Performance of skills that involved turning in the device varied between participants, which was reflected in the relatively lower percentage of achievement of supervision only or less for the basic skill of *Walk a 180° curve* and the advanced skill of *Arrest near a chair, pivot turn and sit down*. Not all participants opted to make use of the “turning assistance” feature of the exoskeleton, with some preferring to walk a steady curve in “walking mode,” while others who were more familiar with the use of knee-ankle-foot orthoses (KAFOs) decided to perform 90-degree pivots in “safe standing mode” by pushing up on the frame with their arms. Given the extra weight of the exoskeleton compared to the KAFO, these alternative techniques to using the turning assistance feature of the exoskeleton proved to require higher assistance from the therapist to maintain stability. This effect was also prevalent in the TUG tests whereby the greatest loss of time and difficulty arose in the 180 degrees turn and the pivot to sit down into the chair.

The most difficult advanced skill identified in this study was to *Enter an elevator, ride to another floor, and exit without requiring the elevator door to be held open*, with 45% of participants requiring moderate assistance to complete this skill by the sixth training block. A previous study with the Indego exoskeleton showed that users with high thoracic paraplegia could enter and exit a lift, but with varying levels of assistance (Hartigan et al., 2015). This is a highly complex task that combines the need for maneuvering and positioning correctly in front of the doors, maintaining standing balance with one hand to call the lift, timely activation of the first step with the device once the doors open to enter/exit the lift, and the performance of a 180 degree turn inside the tight confines of a lift that normally can only fit 1 standard wheelchair.

A unique and unexpected result of post-study data analysis found in all participants in the chronic phase was that they performed consistently better for skill achievement than those in the sub-acute phase, irrelevant of NLI or AIS classification. The three participants in the sub-acute phase were in the early stages of gait training with a KAFO and were still having ongoing issues with fluctuating spasticity, which likely meant they were not able to perform as well as those participants in the more stable chronic phase. This finding is somewhat surprising as we expected NLI to have a higher impact on skill completion than time since injury. It is remarkable that a participant with a cervical motor complete injury in the chronic phase performs better than those with cervical motor incomplete or thoracic motor complete injuries in the sub-acute phase. This finding challenged our preconceptions that people with tetraplegia would perform worse in these tests, and it highlights that potential users with more rostral NLI are not necessarily less suited to training these skills in the ABLE Exoskeleton, especially if they are in the chronic phase of their SCI.

The HST intended to bring the skills completed in training sessions together in a continual sequence to more closely simulate daily life situations, whereby skills are rarely performed independent of one another. Our results of the HST in this study were similar to that by van Dijsseldonk et al. (2017) in terms of the types of tasks involved and the overall layout, however, direct comparability is not possible without the same task layout being used. In our study, LoA for tasks was higher in the HST when compared to block 6 of training, with participants needing moderate assistance or less for nine out of the 10 tasks. This is likely due to the increased difficulty level of completing one task directly after another in the HST, rather than separately as in the training sessions. Additionally, the environment where the test was completed differed from the regular training sessions, which also could have an impact on the results. van Dijsseldonk et al. (2017) similarly reported greater difficulty when skills were completed in a consecutive sequence during their final skills test, with only 75% of the skills being achieved by all participants, further highlighting that exoskeleton skills cannot be only tested as separate items if home or community use is being considered. No patterns in our study were found in participant performance of the HST based on NLI or AIS grade, however, those in the chronic phase performed significantly better than those in the subacute group. Notably, all participants with a chronic SCI were able to *arrest gait on command* with supervision only or less, while all but one participant with chronic SCI were able to *Walk a 90 degree curve, Walk a 180 degree curve, Pass a narrow passage,* and *Walk over a martial arts mat* with supervision only or less. Notably, half of the participants in the study by van Dijsseldonk et al. (2017) were unable to complete the *Walk over a martial arts mat* skill, however, it is very important to note that participants in that study were not provided with any physical assistance.

Significantly improved results for the 10MWT and 6MWT from baseline to end of training demonstrate a positive learning effect from training with the ABLE Exoskeleton, with participants able to walk faster and for longer in the device by the end of the study. Average walking speeds for all participants during the 10MWT improved from 0.12 m/s to 0.17 m/s by S21, however this is slower than the 0.3 m/s recorded in the study with the *ABLEknee* prototype (Wright et al., 2023), with these speeds being below the thresholds for community ambulation. However, it should be noted that in this study a substantial part of the sample were people with tetraplegia and/or a complete SCI. A previous study suggested that a gait speed of >0.49 m/s is required for community ambulation, due to the time needed to cross a pedestrian crossing (Andrews et al., 2010). Studies testing other exoskeletons with participants with chronic, motor complete or incomplete spinal cord injury have achieved speeds of between 0.28 to 0.60 m/s (ReWalk) and 0.36 to 0.38 m/s (Indego) in the final 10MWT (Khan et al., 2019; Tefertiller et al., 2018). However, results of the ReWalk trial were taken after participants had completed >40 training sessions (Khan et al., 2019), while although a comparable 24 training sessions were completed in the Indego trial, the NLI of participants ranged from T4-L2 (Tefertiller et al., 2018). Walking speed results in our study were consistent with another study with the ReWalk exoskeleton in individuals with complete thoracic SCI whereby half of the participants achieved walking speeds of 0.03–0.45 m/s (Esquenazi et al., 2012). If the main objectives of this study had prioritized walking speeds, there might have been more notable advancements, as the training approach would have emphasized these results instead of skill completion with minimum LoA. Still, the results of the present study show that adjustments must be made to the ABLE Exoskeleton to allow the users to achieve the identified speeds for community ambulation.

Results from the device metrics in this study were in keeping with other exoskeleton studies, with increases in the average number of steps (174%) and distance (183%) respectively from session 1 to 21 (McIntosh et al., 2020; Gagnon et al., 2018). The average of 456 steps and 163 m walked per session is lower than that of the study with the previous *ABLEknee* prototype (599 steps, 281 m) (Wright et al., 2023), however, it is important to note that many of the skills attempted during this current trial required the participant to focus on standing balance and more intricate skills such as turning, pivoting or opening doors rather than solely standing and gait training.

Despite Spinal Cord Injury Rehabilitation Evidence (SCIRE) recommending the use of the QUEST 2.0 for evaluation of assistive technology, an international survey among 110 clinicians revealed that most do not use QUEST 2.0 or any other specific outcome measure to evaluate assistive technology (Scivoletto et al., 2019). In our study, the systematic evaluation of participants’ and therapists’ experience with the QUEST 2.0 assessment was key to identifying potential opportunities for improvement of the exoskeleton. The results of the SRQ-GH showed a positive perceived improvement in five out of the seven categories assessed when training with the ABLE Exoskeleton: cardiovascular fitness, musculoskeletal pain, bladder/bowel, spasticity, and sleep quality. By the follow-up assessment, the trajectory of improvement that had been felt by some participants during training did not continue but was maintained at the same level. One participant reported a worsening of spasticity and musculoskeletal pain that had improved during training due to no longer training with the device. These findings let us hypothesize that gait training with the ABLE Exoskeleton may elicit a positive impact on the general health of persons with SCI. As the personal-use exoskeleton will be developed for those no longer in a clinical setting, it is positive that users were able to note a beneficial impact on these areas that are key to the maintenance of general health in the years post-SCI. Nevertheless, more studies need to be conducted to demonstrate this.

Following the conclusion of the trial, five key areas were established as aspects that the engineers at ABLE Human Motion should seek to improve to develop a personal-use exoskeleton that is better suited to a home or community environment:

1. Improve the cooling system for motors to prevent overheating when used with users with high levels of spasticity.
2. Improve Sit-to-stand transition to reduce LoA required for the user.
3. Improve the speed of transitions between exoskeleton states (i.e., turning to walking states).
4. Consider a richer display on the Remote Controller for the end-user.
5. Increase available walking speed to users to improve access to community environments.

This study allowed us to identify these key areas for improvement working in a controlled and safe environment, where the exoskeleton is always supervised by clinical professionals. Next, once the technical developments needed to address those aspects are completed, the new personal-use exoskeleton created by ABLE Human Motion should be tested in the real-world setting: home and community environments.

Some limitations were identified in this study. In both sites, the chronic population who participated in the training were coming on an outpatient basis and had to come regularly to the centers, meaning they had a very high motivation for participation. This might have introduced a recruitment bias. Although identical setups were intended for the training sessions across both centers, there were structural limitations that we could not overcome in each center in regard to the specific specifications of the door and of the elevator for the respective skills they were used for. However, it was ensured that both centers used as similar environments as possible for these skills. Furthermore, we acknowledge that the results of our simulated community environment cannot directly be generalized to a real community setting. Varying levels of experience with the ABLE Exoskeleton between therapists may have led to some therapists being less confident in allowing participants to attempt skills independently. Device metrics of standing time, walking time and distance walked during training sessions were calculated using the software of the ABLE Exoskeleton which has not been externally validated using external equipment, therefore these specific results may be subject to inaccuracies in the software. Furthermore, although the HST is a variation on the Final Skills Test from van Dijsseldonk et al. (2017), neither of these tests have been clinically tested for validity or reliability. Similarly, the SRQ-GH has not been clinically tested for validity or reliability therefore results for these outcome measures should be taken within the context of their direct application here in this study. While our user-centered-design study was aligned to trial protocols of exoskeletons previously tested in end users with mobility impairments, we did not aim for direct benchmarking of results with other trials due to differences in intervention protocols and purposely chosen diversity of participants’ characteristics. For this purpose, the project “EUropean Robotic framework for bipedal locomotion bENCHmarking (EUROBENCH)” funded by the European Union’s Horizon 2020 research and innovation program was created. EUROBENCH provides standardized evaluation schemes which primarily support benchmarking of exoskeletons and in people without disabilities and might enhance the reproduction, replication, and comparison across end user studies in the future (Remazeilles et al., 2022).

## Conclusion

The study results show that the new hip-knee-powered ABLE Exoskeleton is safe, feasible and usable for people with SCI with respect to donning, doffing and the performance of exoskeleton skills. A high rate of success was found in a range of advanced skills needed for the home or community environment, while our results show that the supervised use of the exoskeleton in the clinical environment was a highly valuable tool for identifying the challenging tasks and technological developments for a usable personal-use exoskeleton. Key development points for the next iteration of the personal-use exoskeleton were identified, and once the ideation and design process has worked on these areas, the revised version should be tested again in a real-world setting with persons with SCI to evaluate the device’s general suitability for tasks in the home and community environments and its possibilities toward full participation in society.

## Data Availability

The raw data supporting the conclusions of this article will be made available by the authors, without undue reservation.
