# Supplementary material for: User-centered design of a personal-use exoskeleton: a clinical investigation on the feasibility and usability of the ABLE Exoskeleton device for individuals with spinal cord injury to perform skills for home and community environments
Source: Front Neurosci. 2024 Sep 26;18:1437358. doi: 10.3389/fnins.2024.1437358 (PMC11464447; doi:10.3389/fnins.2024.1437358)
Supplement: Supplementary file 1 [file Table_1.pdf]

Supplementary Material 1: Level of Assistance definitions. Level of Assistance was defined separately for Donning/Doffing of the device and for the basic and advanced skills during training sessions and the HST.

Level of Assistance	Don/Doff	Skill completion
Total assistance	Participant performs 0-25% of the effort to don/doff the exoskeleton.<br>Participant is essentially reliant on the trainer to perform all aspects of the donning/doffing.	Participant performs 0-25% of the effort to use the exoskeleton.<br>Two therapists are required to support the participant in the device at all times.
Maximum assistance	Participant performs 25-50% of the effort to don/doff the device.<br>Participant needs maximum assistance to transfer to device and position legs but may be able to adjust the thigh straps.	Participant performs 25-50% of the effort to use the device.<br>Participant needs maximum assistance from the therapist to remain balanced.
Moderate assistance	Participant performs 50% to 75% of the effort to don/doff the device.<br>Participant needs moderate assistance to transfer to device and position legs but may be able to adjust the thigh and shin straps.	Participant performs 50% to 75% of the effort to use the device.<br>The therapist has both hands on the participant or device at all times to provide occasional guidance or balance support.
Minimal assistance	Participant performs > 75% of the effort to don/doff the device.<br>Participant can transfer to device and adjust straps but may need help to position legs.	Participant performs > 75% or more of the effort to use the device.<br>The therapist has one hand on the participant or device for infrequent guidance or balance support.
Supervision	The therapist is not touching the participant but may provide verbal prompts or contact guarding to ensure safety.	The therapist is not touching the participant but is close enough to provide support for balance or guidance as needed.
Independent	Participant is fully independent donning/doffing device.	The participant is fully independent while using the device and the therapist does not provide any assistance.
