# Supplementary material for: User-centered design of a personal-use exoskeleton: a clinical investigation on the feasibility and usability of the ABLE Exoskeleton device for individuals with spinal cord injury to perform skills for home and community environments
Source: Front Neurosci. 2024 Sep 26;18:1437358. doi: 10.3389/fnins.2024.1437358 (PMC11464447; doi:10.3389/fnins.2024.1437358)
Supplement: Supplementary file 2 [file Table_2.pdf]

Supplementary Material 2. Self-Report Questionnaire on the perceived impact of the use of the device on General Health (SRQ-GH)

We would like to ask you to report your perceived changes in different health-related aspects during the **last 15 days**. Please indicate the impact on the different categories in the following way:

- 0 represents no change at all (e.g. no change of amount or intensity of previous persisted back pain).
- 1 to 3 represents a mild, moderate or maximum positive change (e.g. 1 for a mild reduction of previous persistent back pain)
- -1 to -3 represents a mild, moderate or maximum negative change (e.g. -3 for recently developed severe back pain)

	-3	-2	-1	0	1	2	3
**Cardiovascular health** *(Shortness of breath, Fatigue, Circulation)*	O	O	O	O	O	O	O
If a change, is this related to Exoskeleton training?	YES<br>NO						
**Musculoskeletal pain** *(Joint or muscle pain)*	O	O	O	O	O	O	O
If a change, is this related to Exoskeleton training?	YES<br>NO						
**Neuropathic pain** *(burning pain or abnormal feeling at or below injury level)*	O	O	O	O	O	O	O
If a change, is this related to Exoskeleton training?	YES<br>NO						
**Bladder and bowel** *(accidents, frequency, assistance)*	O	O	O	O	O	O	O
If a change, is this related to Exoskeleton training?	YES<br>NO						
**Skin** *(Pressure injuries, bruising, marking)*	O	O	O	O	O	O	O
If a change, is this related to Exoskeleton training?	YES<br>NO						
**Spasticity** *(Spasms, rigidity)*	O	O	O	O	O	O	O
If a change, is this related to Exoskeleton training?	YES<br>NO						
**Sleep quality** *(number of hours, restlessness)*	O	O	O	O	O	O	O
If a change, is this related to Exoskeleton training?	YES<br>NO						
