# Supplementary material for: User-centered design of a personal-use exoskeleton: a clinical investigation on the feasibility and usability of the ABLE Exoskeleton device for individuals with spinal cord injury to perform skills for home and community environments
Source: Front Neurosci. 2024 Sep 26;18:1437358. doi: 10.3389/fnins.2024.1437358 (PMC11464447; doi:10.3389/fnins.2024.1437358)
Supplement: Supplementary file 3 [file Table_3.pdf]

Supplementary Material 3. Study outcomes. A statistical summary of the study outcome measures at the different assessment points is shown. The p-values correspond to Friedman's test (for multiple measures) or Wilcoxon's signed rank test (for two measures).

Measure	Assessment point	Number of participants assessed (%)	Mean ± SD (Median ± IQR)	Range	*p*-value
**Time to don and doff**					
**Donning time (min:sec)**	Baseline (S1)	10 (100%)	7:59 ± 2:02 (7:28 ± 0:36)	5:51 - 13:11	0.08
	Final training (S21)	10 (100%)	6:18 ± 1:59 (5:57 ± 2:41)	3:00 - 9:55	
**Doffing time (min:sec)**	Baseline (S1)	10 (100%)	3:50 ± 1:36 (3:55 ± 2:26)	1:18 - 5:53	0.05
	Final training (S21)	10 (100%)	2:44 ± 1:03 (2:40 ± 1:31)	1:28 - 4:39	
**Donning + doffing time (min:sec)**	Baseline (S1)	10 (100%)	11:50 ± 3:19 (11:07 ± 3:01)	7:53 - 18:56	**0.03**
	Final training (S21)	10 (100%)	9:02 ± 2:55 (8:46 ± 4:04)	4:43 - 13:51	

Measure	Assessment point	Number of participants assessed (%)	Mean ± SD (Median ± IQR)	Range	*p*-value
**Device usage metrics**					
**Upright time (min)**	Best session within the training block 1	10 (100%)	31.80 ± 10.34 (34.50 ± 11.00)	8 - 44	0.44
	Best session within the training block 6	9 (90%)	32.44 ± 6.15 (30.00 ± 8.00)	24 - 44	
**Walking time (min)**	Best session within the training block 1	10 (100%)	17.10 ± 6.56 (19.00 ± 6.50)	2 - 24	0.09
	Best session within the training block 6	9 (90%)	23.22 ± 5.87 (24.00 ± 8.00)	14 - 33	
**Steps (n)**	Best session within the training block 1	10 (100%)	323.00 ± 186.68 (319.50 ± 193.25)	44 - 704	**0.02**
	Best session within the training block 6	9 (90%)	563.22 ± 301.13 (552.00 ± 390.00)	234 - 1182	
**Distance (m)**	Best session within the training block 1	10 (100%)	108.96 ± 64.88 (99.10 ± 90.78)	11 - 223	**0.04**
	Best session within the training block 6	9 (90%)	198.17 ± 123.23 (195.00 ± 189.00)	73 - 405	

Measure	Assessment point	Number of participants assessed (%)	Mean ± SD (Median ± IQR)	Range	*p*-value
**Gait performance and perceived exertion**					
**WISCI II (0-20)**	Screening	10 (100%)	3.50 ± 3.87 (2.00 ± 6.75)	0 - 9	0.09
	Post-training	10 (100%)	4.80 ± 4.73 (3.00 ± 7.50)	0 - 12	
**TUG (sec)**	Baseline (S1)	10 (100%)	165.70 ± 35.18 (181.00 ± 49.25)	113 - 213	0.13
	Mid-training (S11)	10 (100%)	143.60 ± 53.26 (130.00 ± 57.00)	76 - 240	
	Final training (S21)	10 (100%)	139.70 ± 52.28 (128.00 ± 52.75)	88 - 268	
**10MWT (m/s)**	Baseline (S1)	9 (90%)	0.12 ± 0.05 (0.11 ± 0.07)	0.05 - 0.23	**<0.01**
	Mid-training (S11)	10 (100%)	0.17 ± 0.09 (0.21 ± 0.15)	0.05 - 0.26	
	Final training (S21)	10 (100%)	0.17 ± 0.06 (0.18 ± 0.11)	0.08 - 0.24	
**6MWT (m)**	Baseline (S1)	8 (80%)	45.38 ± 16.71 (38.50 ± 20.25)	28 - 79	**<0.01**
	Mid-training (S11)	10 (100%)	59.40 ± 31.42 (63.50 ± 58.50)	15 - 100	
	Final training (S21)	10 (100%)	58.25 ± 26.84 (57.50 ± 41.12)	16.5 - 93	
**Borg-RPE (6-20)**	Baseline (S1)	9 (90%)	12.33 ± 2.50 (12.00 ± 1.00)	10 - 17	0.46
	Mid-training (S11)	10 (100%)	12.60 ± 2.95 (12.30 ± 3.75)	8 - 18	
	Final training (S21)	10 (100%)	12.20 ± 2.74 (12.00 ± 2.75)	7 - 16	

Measure	Assessment point	Number of participants assessed (%)	Mean ± SD (Median ± IQR)	Range	*p*-value
**Satisfaction and well-being**					
**Q1 &Q2 WHOQOL-BREF Score (2-10)**	Baseline (S1)	10 (100%)	7.80 ± 1.40 (8.00 ± 1.00)	6 - 10	1.0
	Mid-training (S11)	10 (100%)	7.80 ± 1.48 (8.00 ± 1.00)	5 - 10	
	Final training (S21)	10 (100%)	7.70 ± 1.70 (8.00 ± 0.75)	5 - 10	
**QUEST 2.0 Score (0-40)**	Baseline (S1)	10 (100%)	33.30 ± 5.36 (32.50 ± 10.80)	28 - 40	0.52
	Mid-training (S11)	10 (100%)	30.70 ± 5.89 (29.50 ± 5.50)	21 - 40	
	Final training (S21)	10 (100%)	29.60 ± 7.40 (29.50 ± 10.20)	18 - 40	
**PIADS Score (-78 to +78)**	Mid-training (S11)	10 (100%)	20.30 ± 14.21 (18.50 ± 15.25)	0 to +45	0.32
	Final training (S21)	10 (100%)	18.90 ± 16.07 (17.50 ± 18.00)	-4 to +44	
